# Characteristics of right ventricular free wall motion in young patients with ventricular arrhythmia, a cardiac MRI study

**DOI:** 10.1186/1532-429X-14-S1-O108

**Published:** 2012-02-01

**Authors:** Daniel H Katz, Moneal Shah, Ellen Cummings, Jie J Cao

**Affiliations:** 1 Cardiology, St. Francis Hospital, Roslyn, NY, USA

## Background

Right ventricular (RV) regional wall characteristics are important in CMR evaluation for arrhythmogenic RV dysplasia (ARVD). While some features are well defined, others are of uncertain clinical significance, due in part, to the high prevalence of RV wall motion variants found in normal subjects. In this study we performed a retrospective review of RV free wall characteristics in a large cohort of young patients referred for ventricular arrhythmia evaluation and compared the findings with an age-matched normal cohort.

## Methods

The study consisted of 159 consecutive patients aged 18 to 49 years referred for CMR due to ventricular arrhythmias and 51 age-matched normal controls. All subjects underwent CMR. Cine images of the 4-chamber view were reviewed to characterize the RV wall motion as having bulge or outpouching at end systole or at end diastole. The bulges were further classified by location, size and relationship to the moderator band. The size of bulges were graded as small (≤0.5 cm^2^), moderate (0.5 - 1 cm^2^) and large (> 1 cm^2^).

## Results

Mean age was 38 years. Of the 159 patients, 62 (39%) presented with premature ventricular contractions (PVCs) and 97 (61%) with ventricular tachycardia (VT). There was no significant difference in RV size and function between patients and controls. Diastolic bulges were most common in the VT group (10 cases, 10%), rare in PVC and control group (1 case each, 2%). Large diastolic bulges were present only in 6 VT group cases. Systolic bulges were more common, with 11/51 (22%) in normal control, 5/62 (8%) in PVC group and 20/97 (21%) in VT group. All systolic bulges in the control group were small while bulge size was mainly moderate in the PVC group (4/5) and large bulges were only present in the VT group (4/20). All systolic bulges were in close proximity to a moderator band in the control group but 6/34 (18%) were not in the arrhythmic groups. Basal location is uncommon but 5 of 6 cases were found in the VT group, 1 in the PVC group and none in the control group.

## Conclusions

RV characteristics that are predominantly associated with VT include bulges or outpouchings present in diastole, basal location, large size and in the absence of a relation to the moderator band. Our findings suggest that risk stratification of ventricular arrhythmia should consider those RV features particularly in cases where the diagnostic criteria of ARVD are unmet.

## Funding

None.

